# Impact of regional multi-disciplinary team on the management of complex urogynaecology conditions

**DOI:** 10.1007/s00192-023-05513-5

**Published:** 2023-04-10

**Authors:** Hui Ling Ong, Inna Sokolova, Wael Agur

**Affiliations:** 1 University of Glasgow, Glasgow, UK; 2 NHS Ayrshire & Arran, Kilmarnock, UK

**Keywords:** Multi-disciplinary team, Pelvic floor dysfunction

## Abstract

**Introduction and hypothesis:**

Following the publication of the National Institute for Health and Care Excellence guidelines on the management of pelvic floor dysfunction, articles speculating on the benefits and costs of local and regional multi-disciplinary teams (MDTs) have been in circulation. To date, there has been no formal assessment of the impact of a regional MDT on the management of women with complex urogynaecological conditions.

**Methods:**

Throughout the existence of the West of Scotland (WoS) Regional Urogynaecology MDT, from May 2010 to December 2015, 60 patients with complex Urogynaecology conditions were discussed. Data were collected on presenting condition, pre- and post-MDT management plans, and treatment outcomes.

**Results:**

The average age was 52.6 years (range 21–91 years). All meetings had at least 1 urogynaecologist, 1 gynaecologist, 1 reconstructive female urologists, 1 urodynamicist and, on average, 3 continence nurses, 4 physiotherapists, as well as 1 clinical librarian to conduct a literature search and 1 secretary for administrative support. The majority of the referrals dealt with urinary incontinence (*n*=34) and 8 patients presented with mesh complications alongside other pelvic floor disorders. The MDT made changes to the original referrer’s management plan in at least 25 (41.7%) patient presentations. Twenty-two out of all the patients discussed (36.7%) were reported as cured or improved in their condition following the MDT-recommended management.

**Conclusion:**

The WoS Regional Urogynaecology MDT had a positive impact on the management of women presenting with complex condition(s). Cross-sharing of resources between hospitals within the region provided a wider range of management plans, better tailored to each individual.

## Introduction

Pelvic floor dysfunction resulting in urinary incontinence and pelvic organ prolapse affects 17–45% of adult women [1]. These conditions reduce quality of life across all age groups and are linked to childbirth, menopause and advanced age. These conditions account for at least 20% of major gynaecological surgeries in developed countries [2]. Recurrent stress urinary incontinence (SUI) following surgery has been shown to affect 20% of women [3].

The National Institute for Health and Care Excellence (NICE) has published recommendations for local and regional multi-disciplinary teams (MDTs) to be engaged in the management of pelvic floor dysfunction. The regional MDT would include urogynaecologists, urologists, colorectal surgeons, care of the elderly physicians, physiotherapists and specialist nurses. Complex and recurrent pelvic floor dysfunction presentations were advised to be escalated to the regional MDT. Such presentations include women requiring repeat continence surgery, those with co-existing bowel problems or mesh-related problems and those considering primary surgery prior to completing their family. The rationale behind this recommendation is that a regional team would be able to provide expert assessments and access to a broader range of management options while removing the “organ-specific” approach to managing complex presentations [4].

The concept of MDT has been well established in oncology; however, in urogynaecology and urology there are different views on the efficacy or necessity of an MDT. Since the publication of NICE recommendations in 2019, there have been several articles documenting the potential benefits and disadvantages of having MDT meetings in the field of urogynaecology. It has been suggested that MDT meetings enable standardisation of patient care and drives the development and implementation of evidence-based decisions [4]. Additionally, there has been evidence of MDTs improving patient satisfaction, ensuring adequate risk management in the implementation of new procedures and fostering good team-relationships [5]. However, some arguments have also been made regarding the disadvantages of MDTs. Among the main concerns are the high costs, time-consuming discussions, delay in management due to MDT referrals and the efficacy of decisions being made [6, 7]. For example, a survey taken by gynaecologist and urogynaecologist members of the British Society of Urogynaecologists (BSUG) illustrates that a significant proportion disagree with the need for an MDT [8].

The main uncertainty lies in the extent to which the MDT alters the original management plan by the referrer and whether any alterations are associated with better clinical outcomes [9, 10]. There is no reliable evidence in the literature regarding the emerging use of local and regional MDTs in urogynaecology and, apart from the opinions expressed in the NICE guidelines, there is a scarcity of universally accepted standards for multi-disciplinary care. The aim of this study is to evaluate the structure and impact of the West of Scotland (WoS) Regional MDT discussions on the management and outcomes for women presenting with complex pelvic floor dysfunction.

## Materials and methods

The WoS Regional Urogynaecology Multi-disciplinary Team was established in May 2010 and continued until December 2015. The MDT held 21 meetings, at an average rate of one meeting every 3–4 months. The MDT members included urogynaecologists, urologists, video-urodynamicists/ clinical physicists, physiotherapists, specialist nurses, clinical librarians and administrative staff. A colorectal surgeon was invited to the meetings whenever there was a complex lower bowel condition. The meetings were also attended by community continence nurses, urology/gynaecology trainees, sub-specialty trainees and research fellows. The team members attended the lunchtime meetings on their personal good will, with no additional funding.

The purpose of the MDT meetings was to discuss complex urogynaecological and female urological conditions that require sub-specialised multi-disciplinary input at a regional level to provide high quality care to women with complex pelvic floor dysfunction. This is particularly important in the absence of reliable scientific evidence as to how to manage many such complex conditions.

**Fig. 1:**
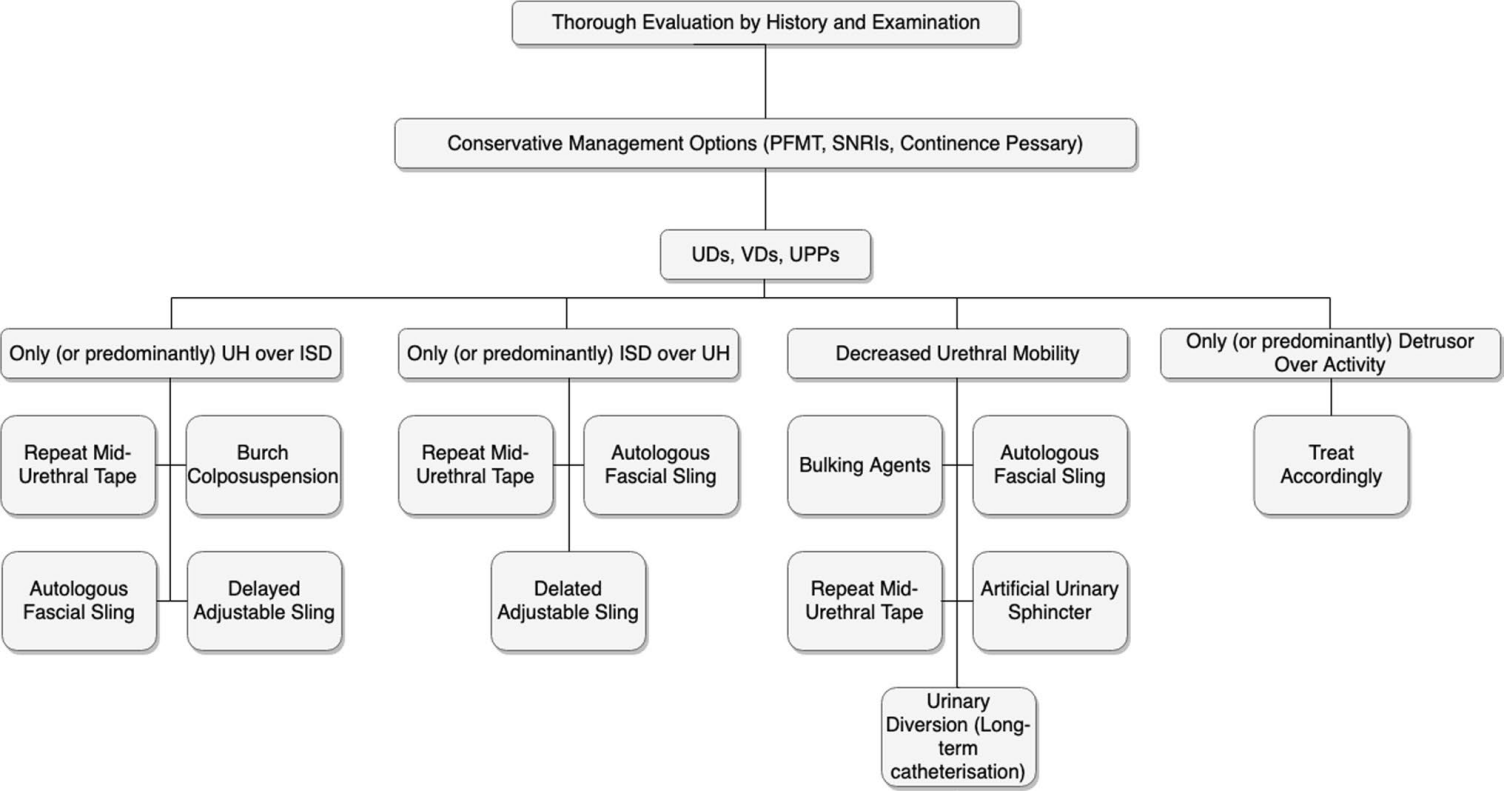
Management of recurrent or persistent stress urinary incontinence following continence surgery. Care pathway followed by the West of Scotland Urogynaecology multi-disciplinary team. *ISD*, *PFMT* pelvic floor muscle training, *SNRI* serotonin and norepinephrine reuptake inhibitors, *UD* urodynamics, *UH* urethral hypermobility, *UPP* urethral pressure profilometry, *VU* video-urodynamics

The referral criteria for the regional MDT included recurrent or persistent stress and/or urgency urinary incontinence, and pelvic organ prolapse following continence and/or prolapse surgery, pelvic mesh complications, previous mesh surgery, suspected Fowler’s Syndrome, considerations of major pelvic surgery during reproductive age and other miscellaneous complex presentations. The general outline of management for recurrent SUI in women followed by the MDT to guide their decisions is outlined by a flow chart (Fig. 1).

The data for all women discussed at the WoS Regional MDT throughout its existence were collected and anonymised. Time between referral, discussion and investigation (e.g. video-urodynamics) and implementation of the management plan were analysed along with the reason for referral, initial management plan, and MDT efficacy indicated by its impact on the original recommendation by referring clinicians. The purpose of the analysis was to give an indication of the time taken for resolution of the cases, the efficacy of the MDT decisions and the impact of the MDT as a whole on clinical outcomes.

All data were analysed using SPSS Statistics (Version 23). As this was a retrospective study aiming to evaluate the Regional MDT service, this study did not require research ethics approval.

## Results

The Regional MDT discussed the complex urogynaecology conditions of 60 women. The average age was 52.62 years (range 21–91 years). On average, meetings were attended by 1 urogynaecologist, 1 gynaecologist, 2 reconstructive urologists, 1 urodynamicist, 3 continence nurses, 4 physiotherapists, 1 clinical librarian and 1 secretary for administrative support.

The mean time between patients being referred to the WoS Regional MDT and the meeting discussion date was 101.45 days or 3 months, with wide variations of duration between 1 day and 10 months. Women who required further investigations in the form of video-urodynamics waited, on average, 203 days (7 months) between MDT discussion and the investigation.

Women who were discussed at the MDTs and progressed to surgical treatment waited for a mean of 263.5 days or 8–9 months before undergoing surgery, whereas women who required investigations done before progressing to surgery waited for 717.4 days or almost 2 years before undergoing surgery. In total, women who did not require video-urodynamics waited 8–9 months before having surgery whereas women who required video-urodynamics waited an average of 2.5 years before having surgery (Table 1).

**Table 1:** Mean number of days between referral, regional multi-disciplinary team (MDT), investigation and surgery

Duration of time between	Mean (SD)	Range
Referral and MDT discussion, *n*=43 days	101.45 (77.954)	1–305
MDT discussion and surgical treatment (if required), *n*=20 days	263.550 (257.274)	3–1,116
MDT discussion and video-urodynamics (if required), *n*=19 days	203.4737 (199.983)	4–806
Video-urodynamics and surgical treatment (if required), *n*=8 days	717.375 (528.3)	68–1,460
MDT discussion and surgical treatment if video-urodynamics was required, *n*=8 days	929.00 (464.560)	387–1,649

**Fig. 2:**
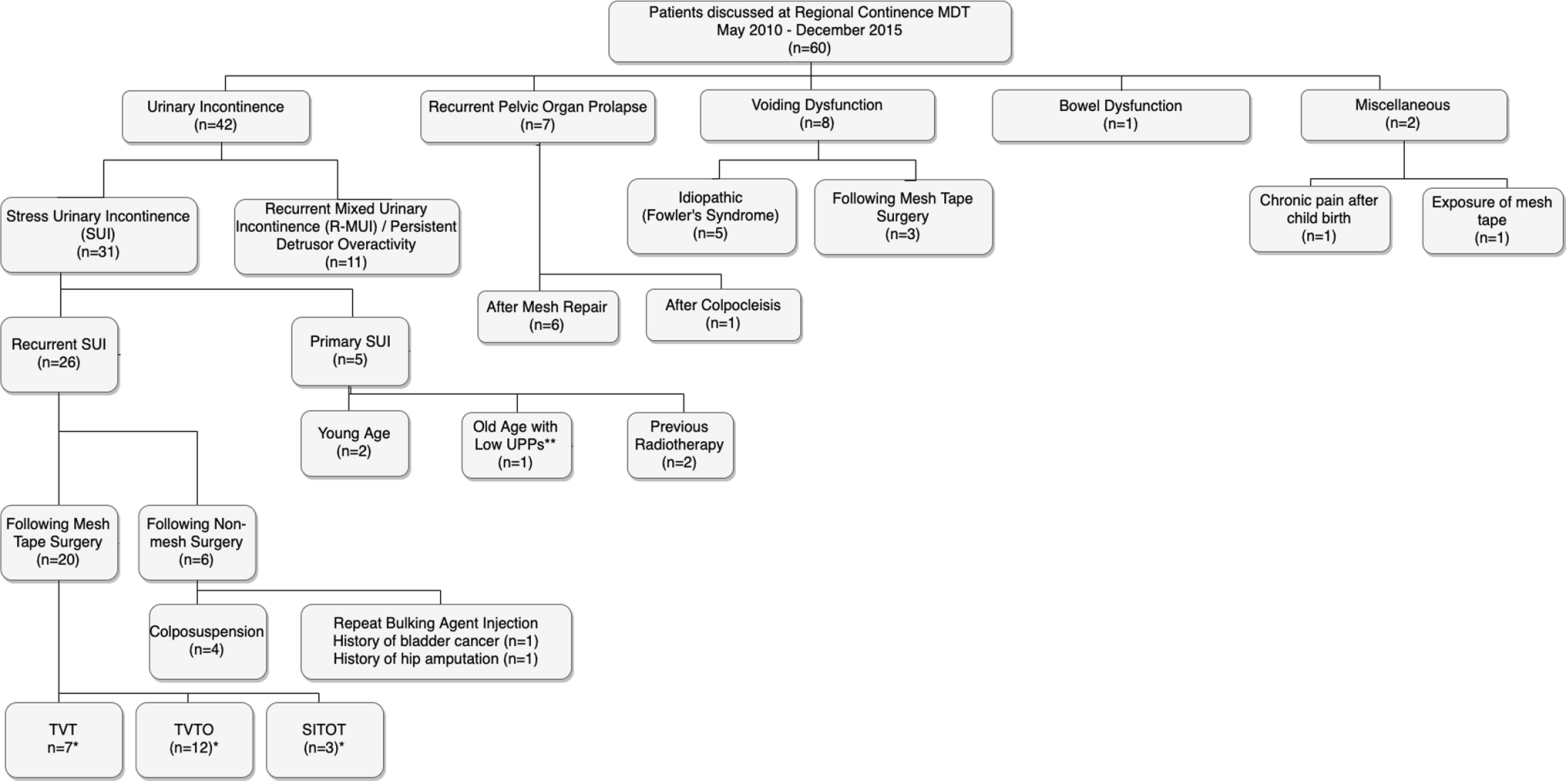
Organisational/distribution chart patients under each referral criterion

Of the 60 patients discussed at the WoS Regional MDT, 35 (58.3%) patients were referred by urogynaecologists, 10 (16.7%) by urologists, 8 (13.3%) by general gynaecologists, 5 (8.3%) by continence nurses and 2 (8.3%) by gynaecology trainees (Table 2). The main or sole presentations were urinary incontinence (*n*=42), recurrent pelvic organ prolapse (*n*=7), voiding dysfunction (n=8), bowel dysfunction (*n*=1), chronic pain after childbirth (*n*=1) and exposure of mesh tape (*n*=1). A total of 31 women presented with stress urinary incontinence (SUI), of whom 26 were experiencing a recurrent condition (Fig. 2).

**Table 2:** Categories of complex cases referred for discussion at the Regional Continence multi-disciplinary team (MDT)

Categories of cases referred to regional continence MDT (*N*=60)	No. of cases (%)	Change in management(%)
Women with mesh complications	8 (13.3)	4 (50.0)
Mesh tape exposure (*n*=8)	1 (12.5)	0 (0.0)
and voiding dysfunction (*n*=8)	2 (25.0)	0 (0.0)
and recurrent SUI (*n*=8)	1 (12.5)	1 (100.0)
and recurrent prolapse (*n*=8)	4 (50.0)	3 (75.0)
Women with complex urinary incontinence	42 (70)	18 (43.9)
Women with complex pelvic organ prolapse	7 (11.7)	2 (28.6)
Women with complex bowel dysfunction	1 (1.7)	1 (100.0)
Women with voiding dysfunction	8 (13.3)	3 (37.5)

The MDT followed a care pathway for women presenting with recurrent stress urinary incontinence following a mid-urethral sling procedure (Fig. 1). Recommendation for a further surgical procedure was largely dependent on the balance between recurrent urethral hypermobility (UH) and the development of intrinsic sphincter deficiency (ISD; Fig. 1).

Twenty-six women with recurrent SUI were discussed, 20 following failed mid-urethral sling surgery and 6 followed failed native tissue surgery (Fig. 2). Two patients developed recurrent SUI after two previous mid-urethral sling procedures. Five women presenting with primary SUI required discussion at the regional MDT owing to age extremes or previous pelvic radiotherapy (Fig. 2).

Among the women included in the study, 7 women presented with mesh tape complications, such as chronic pain and/or (worsening) voiding dysfunction, independent of other pelvic floor disorders (Table 2) and 1 woman presented with mesh tape exposure. Five were recommended to undergo mesh removal surgery by the regional MDT, 1 total and 4 partial removal surgery. The recommendation for total removal surgery was for a patient who presented with chronic pain; however, her symptoms persisted. Partial removal surgery was indicated in women with mesh exposure and/or voiding dysfunction, in the absence of chronic pain. Of the 4 partial removal procedures, only 1 patient stated an improvement by 30% and the others had on-going problems at the time.

After MDT discussions, 19 patients (31.7%) were referred for further investigations (video-urodynamics) prior to any other medical intervention or further discussion at a later MDT. Fourteen of the 19 women required more than one MDT discussion for on-going complex conditions and repeated treatments at various stages in time.

**Fig. 3:**
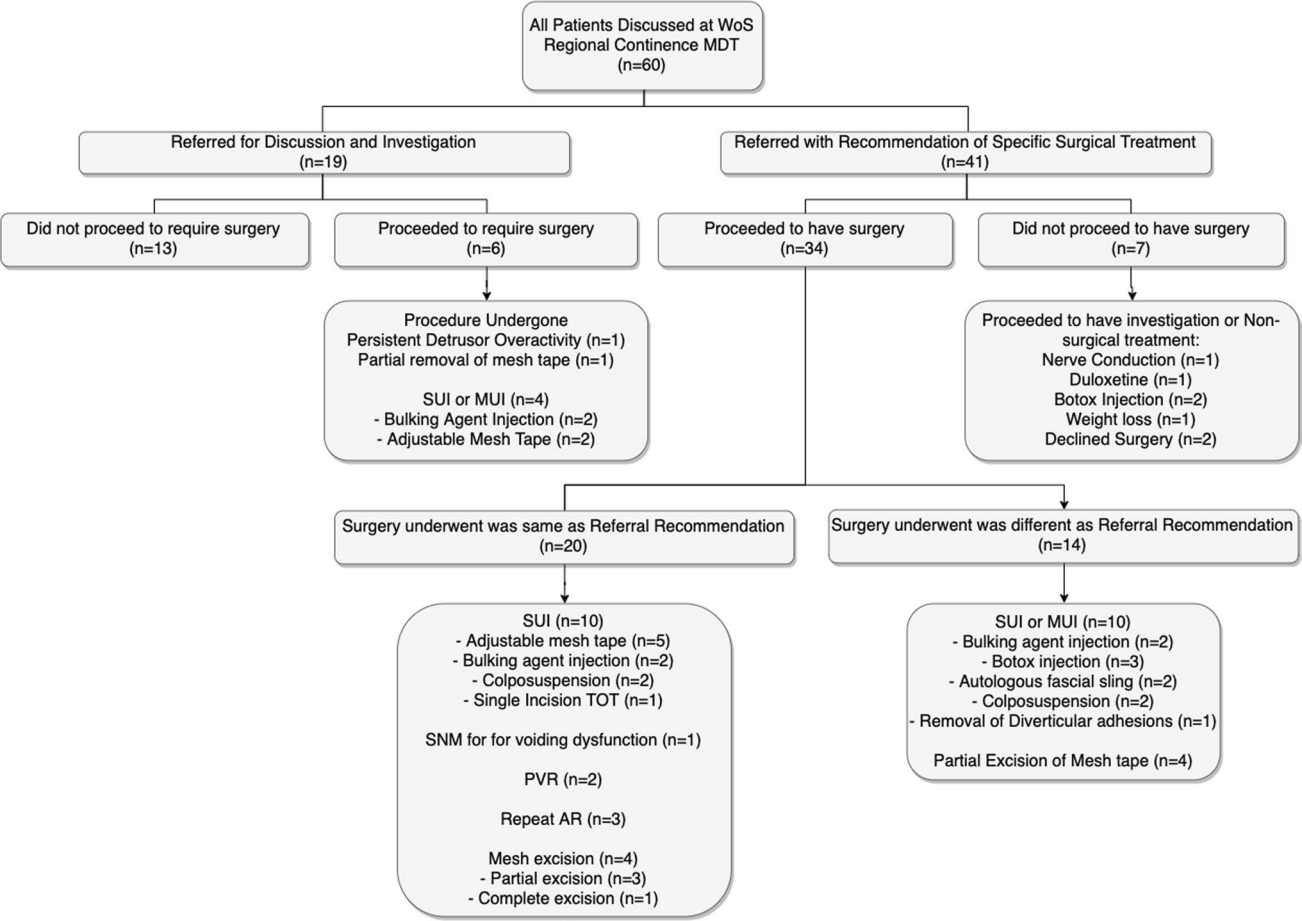
Impact of multi-disciplinary team discussion on the original decision

Twenty-five patients (41.7%) had their management plans altered during MDT meetings (Fig. 3) and 22 patients (36.7%) were determined to be either cured or experience an improvement in their overall health 3 months after carrying out the MDT management plans. Twenty-three patients’ outcomes were not recorded as they were lost to follow-up (38.3%; Table 3).

**Table 3:** Number of patients who required further management/investigations after initial multi-disciplinary team (MDT) discussion and general outcomes

Management of patients, *N*=60	Frequency	Percent
Patients who required multiple MDTs	14	23.3
Patients who required video-urodynamics	19	31.7
Patients whose management plans were altered by the MDT	25	41.7
Patients whose surgical plans were altered by the MDT (*n*=28)	12	42.9
Patient who indicated an improvement in their condition 3 months after treatment	22	36.7
Patients whose outcomes were not recorded/lost to follow-up	23	38.3

## Discussion

### Principal statement

This study demonstrates that Regional MDTs are both feasible and useful to patient care. The Regional urogynaecology MDT has made an impact on the health care provided to women with complex urogynaecology and female urological conditions in the WoS. The MDT recommended a change in the original management plan by the referring clinician for 25 (41.7%) patients. Among the 28 patients who required surgery, the MDT recommended alternative surgical treatment in 12 patients (42.9%). MDT meetings were also a gateway to more invasive testing, including video-urodynamics and urethral pressure profiles.

The regional WoS meetings took place prior to the relevant NICE recommendations in April 2019. The composition of our team largely matches NICE recommendations. The conditions discussed by our group mostly matched the referral criteria recommended by NICE. The majority of women presented with either recurrent stress incontinence and/or mesh complications following continence surgery.

### Strengths of our study

Prior to the publishing of the relevant NICE guidelines, our WoS group pioneered regional MDTs for pelvic floor dysfunction. To our knowledge, our study is the first to demonstrate the usefulness of Regional MDT meetings in health care provided to women with complex urogynaecological conditions. Our study is in line with the results of the Cambridge Study [10] in demonstrating the usefulness of MDT meetings in a local and regional context. In the field of oncology, findings suggest that MDT meetings for non-metastatic cases improved the health of elderly patients after discharge, reduced medication variance and improved follow-up.

### Weaknesses of our study

This was a retrospective analysis of prospectively collected data. Despite the advantage of a long-term follow-up of women whose conditions were discussed at the Regional MDT, a significant number of patients were lost to follow-up data (23 patients, 38.3%). These patients were not counted as failures in our study, and it is likely that, at least in some cases, the pelvic floor condition had been managed locally and no longer required complex interventions by an MDT at a regional level. We recommend keeping a prospective database and contemporaneous follow-up by the administrative team member (or referring clinician). This will enhance the follow-up rate and will also determine whether any loss to follow-up is due to an ongoing need for local (rather than regional) care, movement out of the area, or other reasons.

In addition, we did not use a validated outcome measure in determining the degree of improvement in patient condition following treatment recommended by the Regional MDT. Instead, we relied on clinician reporting of improvement.

Our study demonstrated that referral to video-urodynamics had led to exceptionally longer waiting times, which had inevitably lengthened the referral to treatment pathway.

### Clinical applications

We provide a model for a Regional MDT that will fulfil the NICE criteria set forward in 2013 and confirmed in 2019. Members of the MDT consistently attended the regional meetings and our model provides a draft plan consistent with the NHS England Commissioning Group [11]. The challenges ahead include resources, particularly administrative support, and allocating time for MDT meetings in individual professional job plans. The presence of a clinical librarian provided the evidence-based support for any scientific uncertainty, formulating research questions, conducting the literature search and circulating to the MDT members. The “clinical librarian initiative” of NHS Scotland provided the principles upon which MDT participation of our librarian was established. With no additional funding, the initiative allowed an “embedded” librarian to join frontline clinical staff, as a knowledge mobiliser, in ward rounds and MDT meetings.

Despite these benefits of having a regional MDT, the practice was discontinued in December 2015. This was for several reasons, including changes in the job plans of health care professionals, as it was conducted on the good will of team members, with no funding. Team members were bringing significant information to the table and were keen to improve the care of their patients.

The last WoS Continence MDT meeting took place in December 2015, after 5.5 years of service and 18 months following the suspension of mesh procedures in Scotland in June 2014. In addition, the centralisation of management of mesh complications in a national, Scotland-wide MDT has gradually reduced the need for an alternative/additional regional setup. The development of national care pathways has also reduced the need for individual discussion during a formal regional MDT setting.

The gradual decline in pelvic mesh procedure since 2012, and the subsequent suspension in 2014, had reduced the number of women fulfilling the referral criteria owing to mesh complications, such as erosion, chronic pain and voiding dysfunction. Although the total number of continence procedures has reduced, the native tissue continence and prolapse surgery continue to rise and professionals dealing with complex pelvic floor conditions expect to be presented with new challenges. In our view, these would be best addressed in a holistic multi-disciplinary approach. Therefore, a Regional MDT remains a useful service for women with complex urogynaecological conditions and should be appropriately funded and commissioned.

Despite the recommendation by NICE in 2013, that every patient should be discussed at MDT meetings prior to continence surgery [4], many clinicians remained uncertain about the impact of MDT discussion on patient care [9]. In 2019, NICE continued in this line of recommendation for prolapse surgery by suggesting that all women were to be discussed at the local MDT. NICE also recommended the establishment of a Regional MDT to discuss highly complex cases including mesh complications. Although commissioning/implementation is decentralised, the NHS specialised service specifications [12] stated “*The Regional MDT is central to providing high quality care for women requiring treatment of complex prolapse and urinary incontinence conditions*”.

Transvaginal mesh procedures, for both stress urinary incontinence and pelvic organ prolapse, remain banned in Scotland and the rest of the United Kingdom [13]. However, in countries where mesh procedures (midurethral sling and mesh sacropexy) are still permitted, a regional MDT could be invaluable in reducing any mesh-related harm by confirming patient selection, ensuring that the alternative treatment options are offered and standardising the information on procedure-related risks.

### Recommendation for future successful regional MDT working

Our experience with a regional urogynaecology MDT suggests that adequate administrative support might be the key to its success, as well as appropriate allocation of time in job plans for health care professionals to be able to prepare, attend and act upon the discussions of the MDT meetings. Regional MDTs offer excellent opportunities to train junior doctors. Following the recommendation for regional MDT by NICE, the Independent Medicines and Medical Devices Safety Review (IMMDSR, Cumberlege) and the NHS specialised commissioning, our model could be used to draft a care pathway for a referral to regional MDTs.

## Conclusion

The WoS Regional Urogynaecology MDT had an impact on patient management. Cross-sharing of resources between hospitals within the region provided a wider range of management plans, better tailored to each individual.
